# Diamond and methane formation from the chemical decomposition of polyethylene at high pressures and temperatures

**DOI:** 10.1038/s41598-021-04206-7

**Published:** 2022-01-12

**Authors:** E. B. Watkins, R. C. Huber, C. M. Childs, A. Salamat, J. S. Pigott, P. Chow, Y. Xiao, J. D. Coe

**Affiliations:** 1 Materials Physics and Applications Division, Los Alamos National Laboratory, Los Alamos, NM 87545 USA; 2 Shock and Detonation Physics, Los Alamos National Laboratory, Los Alamos, NM 87545 USA; 3 Department of Physics and Astronomy, University of Nevada Las Vegas, Las Vegas, NV 89154 USA; 4 HPCAT, X-Ray Science Division, Argonne National Laboratory, Argonne, IL 60439 USA; 5 Theoretical Division, Los Alamos National Laboratory, Los Alamos, NM 87545 USA; 6 Present Address: School of Engineering, Case Western Reserve University, Cleveland, OH 44106 USA

**Keywords:** Condensed-matter physics, Chemical physics

## Abstract

Polyethylene (C_2_H_4_)_n_ was compressed to pressures between 10 and 30 GPa in a diamond anvil cell (DAC) and laser heated above 2500 K for approximately one second. This resulted in the chemical decomposition of the polymer into carbon and hydrocarbon reaction products. After quenching to ambient temperature, the decomposition products were measured in the DAC at pressures ranging from ambient to 29 GPa using a combination of x-ray diffraction (XRD) and small angle x-ray scattering (SAXS). XRD identified cubic diamond and methane as the predominant product species with their pressure–volume relationships exhibiting strong correlations to the diamond and methane equations of state. Length scales associated with the diamond products, obtained from SAXS measurements, indicate the formation of nanodiamonds with a radius of gyration between 12 and 35 nm consistent with 32–90 nm diameter spherical particles. These results are in good agreement with the predicted product composition under thermodynamic and chemical equilibrium.

## Introduction

Polyethylene is a broadly used polymeric material with a simple molecular structure comprised of long chains of C_2_H_4_ monomers, making it an important model system for fundamental polymer science studies. Due to strong covalent bonding along the chain and weak van der Waals interactions between chains, polyethylene is relatively incompressible along the chain axis and highly compressible perpendicular to the chain axis. Types of polyethylene, exhibiting a range of material properties, can be categorized by polymer molecular weight and degree of branching. For example, low density polyethylene (LDPE) has a highly branched structure resulting in a density of ~ 0.92 g/cm^3^ and degrees of crystallinity ranging from 40 to 55% while high density polyethylene (HDPE) is primarily composed of linear C_2_H_4_ chains and possesses a higher density (~ 0.94 g/cm^3^) and degree of crystallinity (> 70%). At ambient temperature, HDPE remains chemically stable up to at least 40 GPa with crystalline domains transitioning from an orthorhombic structure to a monoclinic phase above 14 GPa^1^. While stable to extremely high pressures at ambient temperature, in an inert atmosphere polyethylene thermally decomposes to complex mixtures of graphitic carbons and hydrocarbon waxes, oils, and gases at ambient pressure (P) and temperatures (T) of 500–1000 K^2^. High pressure and high temperature decomposition of organic hydrocarbons was first reported by Wentorf where, in the case of polyethylene, diamond products were recovered from decomposition at 15 GPa and 2000 K for 15 min^3^.

While there is little experimental work on the high pressure and temperature decomposition of polyethylene, the decomposition of alkanes ranging from methane (CH_4_) to nonadecane (C_19_H_40_) has been studied under static compression conditions^4–6^. Methane, with a H:C ratio of 4, has a rich phase diagram with several crystalline phases including five cryogenic solids (II-VI) forming only below 150 K and multiple solid phases existing at ambient temperature and high pressures (I, A, B, HP)^7^. Under compression at ambient T, methane transforms from a fluid to FCC phase I at 1.5 GPa, to rhombohedral phase A at 5.4 GPa, followed by a transition to simple cubic phase B between 9 and 18 GPa exhibiting a high degree of hysteresis. Above 25 GPa, phase B undergoes a slight modification to the simple cubic HP phase. Laser heated DAC experiments on methane revealed the formation of diamond reaction products at pressures of 10–50 GPa and temperatures of 2000–3000 K as well as double and triple bonded carbon or hydrocarbon forms^4^. Subsequent studies, conducted over a 10–80 GPa pressure range, showed methane dimerization to ethane above 1100 K, polymerization to longer chains above 2000 K, followed by diamond formation above 3000 K with all reactions exhibiting a weak pressure dependence^6^. Additionally, methane decomposition under dynamic shock loading conditions was observed at pressures and temperatures above 23 GPa and 2300 K^8^. Electrical conductivity measurements under these conditions suggested the formation of diamond-like carbon and hydrogen products^9^. Diamond formation has also been observed from decomposition of longer chain alkanes (C_8_H_18_ to C_19_H_40_) at pressures between 10 and 20 GPa and T ~ 3000 K^5^. In these studies, increasing chain length resulted in higher diamond yields. Notably, C_19_H_40_ has an H:C ratio of ~ 2.1, which approaches that of polyethyelene (H:C ratio = 2).

Although there is limited data on polyethylene decomposition under static high pressure conditions, investigations of polyethylene under dynamic shock loading have been performed using both gas gun driven impactors and laser drives. Under the assumption of full thermodynamic and chemical equilibrium, EOS modeling of HDPE’s shock driven decomposition products predicts the formation of equal mole fractions of diamond and methane at 25 GPa and the gradual replacement of methane with hydrogen for increasing pressures^10^. Experiments using gas gun driven impactors, which maintain high P–T conditions for hundreds of nanoseconds, observed a shock decomposition threshold of 25 GPa and products recovered from shocks reaching 28–40 GPa consisted of methane, hydrogen gas, and carbon soot^11^. Properties of the soot were inconsistent with either graphite or diamond. In contrast, laser driven shock experiments, which maintain high P–T conditions for a few nanoseconds, observed no decomposition products and the retention of crystalline polyethylene at pressures up to 200 GPa^12^. While both shock drives probe the material in conditions far from equilibrium, these results suggest a strong kinetic influence on polyethylene decomposition. Relative to dynamic compression, material in a heated DAC experiences high P–T conditions for orders of magnitude longer times and, in the case of polyethylene decomposition, likely influences the product mixture observed. Here, we report on the decomposition of HDPE at near equilibrium high pressure and temperature conditions and the products recovered after quenching to ambient temperature.

## Results

**Figure 1:**
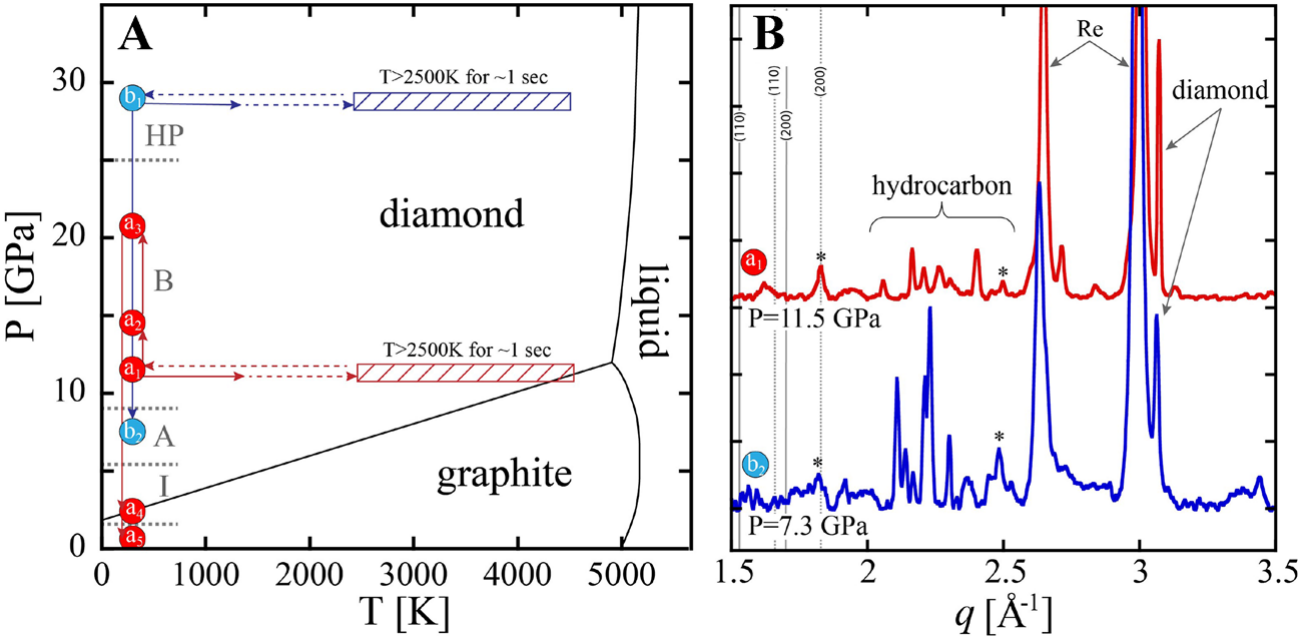
(**A**) Pressure and temperature trajectories for two decompositions of HDPE relative to the carbon phase diagram^13, 14^. HDPE was initially compressed to either 11.5 GPa (‘a’ series, red) or 29.0 GPa (‘b’ series, blue) before heating to decomposition at T > 2500 K for ~ 1 s. Following decomposition, ambient T XRD and SAXS measurements were performed over a series of pressures, denoted by circles a_1_-a_5_ (red) and b_1_-b_2_ (blue). Horizontal dashed lines indicate the ambient T phase boundaries for solid methane crystal structures I, A, B, and HP^1^. (**B**) Representative XRD patterns showing peaks corresponding to diamond and hydrocarbon products after heating HDPE to > 2500 K at 11.5 GPa (red, a_1_) and after heating to > 2500 K at 29.0 GPa and subsequent pressure release to 7.5 GPa (blue, b_2_). The two high intensity peaks correspond to the Re gasket and stars denote peaks corresponding to ruby. For reference, positions of HDPE (110) and (200) peaks at ambient conditions (solid lines) and at P = 15 GPa, T = 300 K (dashed lines) are at lower *q* than the peaks assigned to hydrocarbon products^15^.

HDPE was decomposed at high pressure and temperature in a diamond anvil cell (DAC) heated with a CO_2_ laser. Samples were initially compressed to 11.5 or 29.0 GPa at ambient T, heated to 2500–4500 K for approximately 1 s to decompose the polymer, and returned to ambient T before performing a series of x-ray diffraction (XRD) and small angle x-ray scattering (SAXS) measurements. Due to the inability to obtain sufficiently high quality black-body radiation measurements during the short duration laser heating process, there is a large degree of uncertainty in the estimated temperatures. Approximate P-T trajectories of the experiments are overlaid on the carbon phase diagram in panel A of Fig. 1 and indicate that, for the decomposition conditions studied here, diamond is the equilibrium phase for carbon^13, 14^. Following laser heating, a series of x-ray measurements at the ambient T quenched conditions spanned pressures corresponding to a range of methane phases from fluid to crystalline A, B and HP phases^15^. Representative XRD patterns are shown in panel B of Fig. 1 exhibiting several peaks at 2.0 > *q* > 2.6 Å^−1^, an additional peak at *q* ~ 3.1 Å^−1^, as well as diffraction corresponding to the Re gasket and internal ruby P calibrant. Diffraction from HDPE, either at ambient or elevated pressures, was not detected in these experiments indicating that chemical decomposition of the sample had occurred. The observed diffraction is interpreted as originating from crystalline reaction products formed from decomposition of HDPE with the peak at *q* ~ 3.1 Å^−1^ consistent with diffraction from the cubic diamond (111) plane and the lower *q* peaks consistent with diffraction from molecular crystals of small hydrocarbons.

### XRD from solid carbon products

**Figure 2:**
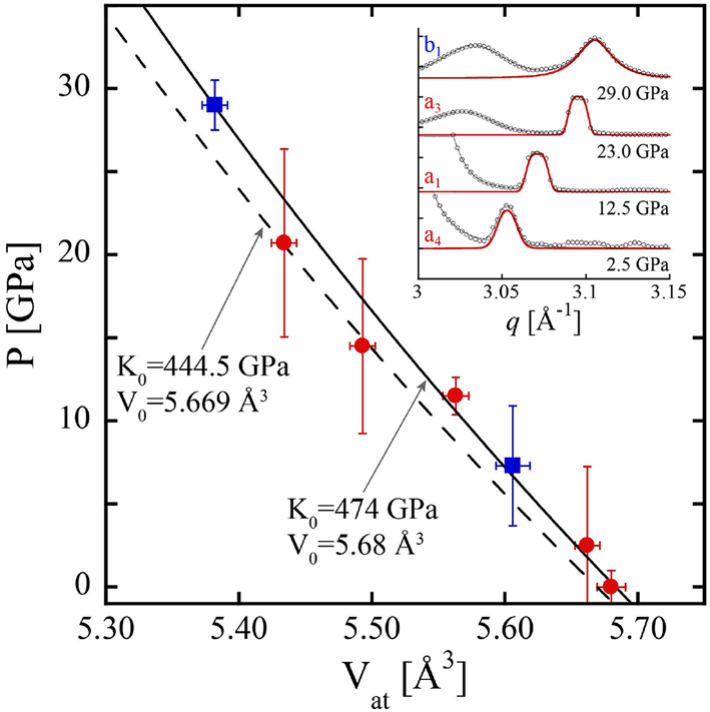
Atomic volume, obtained from the position of the (111) diffraction peak, of diamond products as a function of pressure. Red circles correspond to ‘a’ series measurements (decomposition at 11.5 Gpa) and blue squares to ‘b’ series measurements (decomposition at 29.0 GPa) shown in Fig. 1. The dashed line is the Vinet EOS for cubic diamond from^19^. The solid line is a Vinet EOS fit to the data shown here. Representative fits (red lines) to the diamond (111) diffraction peaks are shown in the inset.

In all XRD measurements, a diffraction peak was observed between *q* = 3.050 Å^−1^ and *q* = 3.106 Å^−1^ (Fig. 2, inset). At ambient pressure, the measured peak position of *q* = 3.050 ± 0.002 Å^−1^ corresponds to a d-spacing of 2.060 ± 0.0015 Å which is in excellent agreement with the 2.0593 Å d-spacing of the (111) plane of cubic diamond^16^. Diffraction from the cubic diamond (220) or (311) planes were outside the *q* range probed by the experiment and there is significant ambiguity associated with the assignment of a product phase based on a single diffraction peak. Alternatively, the peak may be interpreted as diffraction from the graphite (101) plane with a *q* position of 3.0995 Å^−1^ at ambient pressure^16^. However, other peaks associated with graphite, including the low *q* peak originating from the graphite interlayer spacing, were not observed. Additionally, the significantly lower *q* position of the peak relative to diffraction from the graphite (101) plane corresponds to a highly expanded graphite form. Assuming expansion along the more compressible c-axis, the graphite interlayer spacing would have to swell by more than 20%, from 3.348 Å to 4.125 Å, to match the measured ambient pressure measurement. This is significantly larger than the 3.8 Å spacing observed for highly curved and disordered carbon forms such as glassy carbon and carbon onions^17, 18^.

Further support for the assignment of the *q* ~ 3 Å^−1^ peak to diamond products was obtained from the pressure dependence of the diffraction peak position. Assuming a cubic diamond structure, the atomic volume of carbon as a function of pressure obtained from XRD shows a strong correlation with the equation of state (EOS) for cubic diamond (Fig. 2)^19^. Parameters for the Vinet EOS from^19^ are V_0_ = 5.6693 ± 0.0016 Å^3^, K_0_ = 444.5 GPa (fixed), and K_0_’ = 4.18 ± 0.15 where V_0_ is the atomic volume, K_0_ is the bulk modulus, and K_0_’ is the bulk modulus pressure derivative at ambient conditions and reported errors correspond to a 95% level confidence interval^20^. Fixing K_0_’ to 4.18, an orthogonal distance regression (ODR) algorithm was used to fit the P–V data yielding parameter values of V_0_ = 5.683 ± 0.005 Å^3^, and K_0_ = 474 ± 15 GPa with error bars corresponding to one sigma uncertainties^21^. Relative to the literature EOS, the EOS fit to the data resulted in a 2% greater value of V_0_ and 7% greater value of K_0_, slightly outside of the standard errors of the fitting approach. Notably, the bulk modulus of graphite is approximately three times too small to account for the measured pressure dependence of the diffraction peak.

In contrast to the pressure dependence of the peak position, no trend was apparent in the width of the *q* ~ 3 Å^−1^ peak as a function of pressure. However, there were systematic differences in the peak widths associated with the pressure at which decomposition occurred. In the case of HDPE decomposed at 11.5 GPa (Fig. 1, a1–a5), after correcting for instrumental resolution the mean intrinsic full width half maximum (FWHM) of the peak was 0.0092 ± 0.0005 Å^−1^. For HDPE decomposed at 29.0 GPa (Fig. 1, b1–b2), the mean intrinsic FWHM was 0.0199 ± 0.0012 Å^−1^. Assuming a spherical shape and a cubic lattice, the Scherrer formula (*D* = 6.96/FWHM) can be used to estimate the diameter of the crystallites as *D* = 75.3 ± 30.0 nm for decomposition at 11.5 GPa and *D* = 34.6 ± 8.4 nm for decomposition at 29.0 GPa^22^.

### XRD from hydrocarbon products

**Figure 3:**
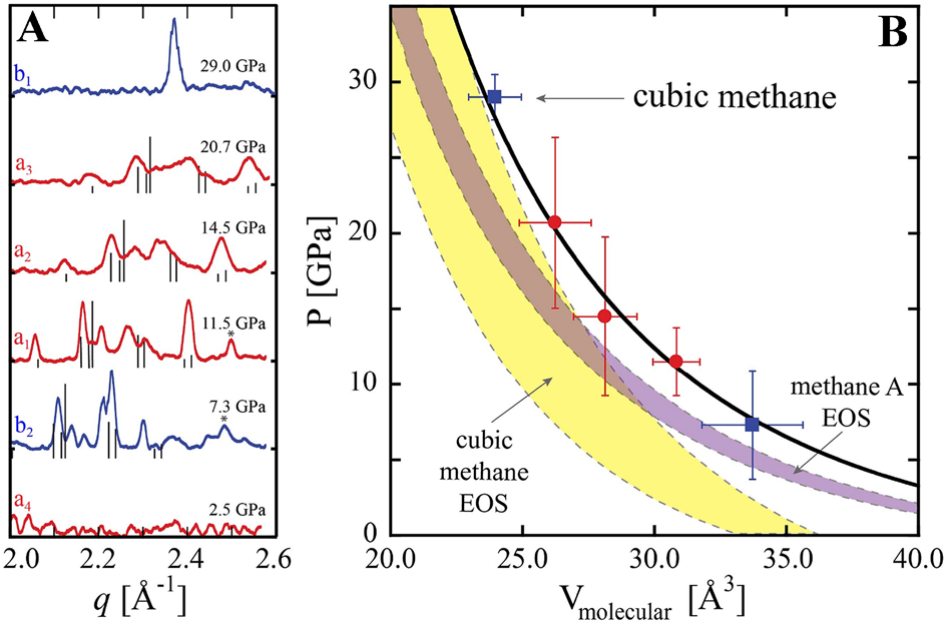
(**A**) Methane XRD patterns as a function of pressure. Red lines correspond to decomposition at 11.5 GPa and blue lines to decomposition at 29.0 GPa. From 7.3 to 20.7 GPa multiple peaks were indexed to methane’s rhombohedral phase A. At 29.0 GPa a single peak was observed consistent with the simple cubic HP methane phase. (**B**) Methane molecular volume, obtained from XRD peak positions, as a function of pressure. Methane A and cubic methane equations of state are shown in purple and yellow respectively, with the width of the curve representing uncertainty^23^. The solid line is the best fitting EOS to the methane A data shown here (P < 25 GPa).

XRD results from HDPE decomposition indicate the formation of cubic diamond products. However, the (C_2_H_4_)_n_ reactant composition requires the existence of other decomposition products consisting of either hydrogen (H) or hydrocarbons (CH). A diffraction signature from additional decomposition products composed of several distinct peaks was observed in the range of 2.0 < *q* < 2.6 Å^−1^ at pressures between 7.3 and 20.9 GPa (Fig. 3A). In the 11.5 GPa decomposition (Fig. 3A, red), these diffraction peaks shifted to higher *q* with increasing pressure (up to 21 GPa) indicating a volume decrease. While the cubic diamond peak remained after releasing pressure to 2.5 GPa, the low *q* peaks were no longer observed suggesting that the associated product was no longer in a solid crystalline state. This observation is consistent, within experimental errors, to the melt line of methane and a transition to a fluid phase below 1.5 GPa at ambient temperature^7^. In the higher pressure HDPE decomposition case (Fig. 3A, blue), a single low *q* peak was observed at 29.0 GPa, suggesting a higher symmetry crystal structure, and after releasing pressure to 7.3 GPa multiple diffraction peaks were observed indicating a transition to the lower symmetry phase. Such a change is consistent with the transition from a high pressure simple cubic methane phase to a lower pressure rhombohedral methane phase (e.g. phase A)^23, 24^.

We considered multiple potential H and CH product species to explain the observed diffraction signal at low *q*. For example, diffraction from crystalline H_2_ at 5 GPa exhibits peaks in the range of 2.7 < *q* < 3.1 Å^−1^, significantly higher in *q* than the peaks observed here^25^. Although this rules out assignment of the observed peaks to pure hydrogen products, it does not eliminate the possibility of H_2_ in the product mixture due to the difficulty detecting hydrogen’s weak diffraction signal. This suggests that the observed diffraction originates from hydrocarbon species in the product mixture. While a wide variety of hydrocarbon bonding arrangements (e.g. linear, branched, cyclic) and molecular weights may be considered, in general molecular crystals composed of higher molecular weight hydrocarbons possess a larger unit cell and lower *q* peaks. For example, at 5.9 GPa molecular crystals of ethane (C_2_H_6_) exhibits two of strong diffraction peaks at q = 1.745 Å^−1^ and q = 2.108 Å^−1^ and crystals of propane (C_3_H_8_) exhibit a set of strong diffraction peaks in the range of 1.485 < *q* < 2.016 Å^−1^^26, 27^. In both of these cases, the dominant diffraction signal is too low in *q* to correspond to the peaks observed here. As a result, we consider methane (CH_4_) to be the most likely crystalline product associated with the observed diffraction. While higher molecular weight hydrocarbons cannot be excluded, following the P drop from 29.0 to 7.3 GPa the transformation of a single diffraction peak to the set of low *q* peaks indicates that significant contributions from other crystalline product species are unlikely.

The dominant diffraction from the methane phase A structure, obtained at 9.1 GPa, consists of eight peaks in the 2.0–2.6 Å^−1^ *q*-range, qualitatively similar to the peaks observed here^15^. In contrast, diffraction from methane phase B at 8.3 GPa consists of only four significant peaks in the same *q* space^28^. While the XRD patterns measured between 7.3 and 20.9 GPa do not directly match the phase A structure, differences may be attributed to peak shifts due to uniaxial compression conditions or to coexistence of methane phase B resulting from the gradual A to B phase transition. Measured diffraction peaks were fit using pseudo-Voigt functions and peak positions were indexed to the eight highest intensity reflections from the phase A structure^15^. Due to small peak shifts likely originating from uniaxial compression, the unit cell volume was calculated for each indexed peak independently using a rhombohedral unit cell consistent with the phase A structure (a = b = c, α = β = γ = 89.45°). The molecular volume was estimated as the mean of the unit cell volumes divided by 21, the number of molecules in the phase A unit cell, with uncertainty corresponding to the standard deviation. Alternate indexing assignments were considered under the assumption that one of the measured peaks originated from the coexistence of phase B and the indexing that yielded the smallest standard deviation of volumes was chosen. For the 29.0 GPa measurement, the single peak was indexed as the simple cubic (300) lattice reflection, corresponding to the dominant diffraction peak observed in^23^, and assigned an uncertainty in molecular volume comparable to the lower P measurements of ± 1 Å^3^. The resulting molecular volume as a function of P is shown in Fig. 3B and is consistent with the previously reported Birch-Murnaghan EOS of methane phase A with parameters V_0_ = 47.1 ± 1.0 Å^3^, K_0_ = 7.85 ± 0.1 GPa, and K_0_’ fixed at 4^23, 29^. An ODR fit to the P–V data below 25 GPa yielded Birch-Murnaghan parameter values of V_0_ = 50.4 ± 8 Å^3^, and K_0_ = 8.3 ± 5 GPa with K_0_’ fixed at 4. Error bars correspond to one sigma uncertainties in the fit parameters.

### Small angle x-ray scattering (SAXS)

**Figure 4:**
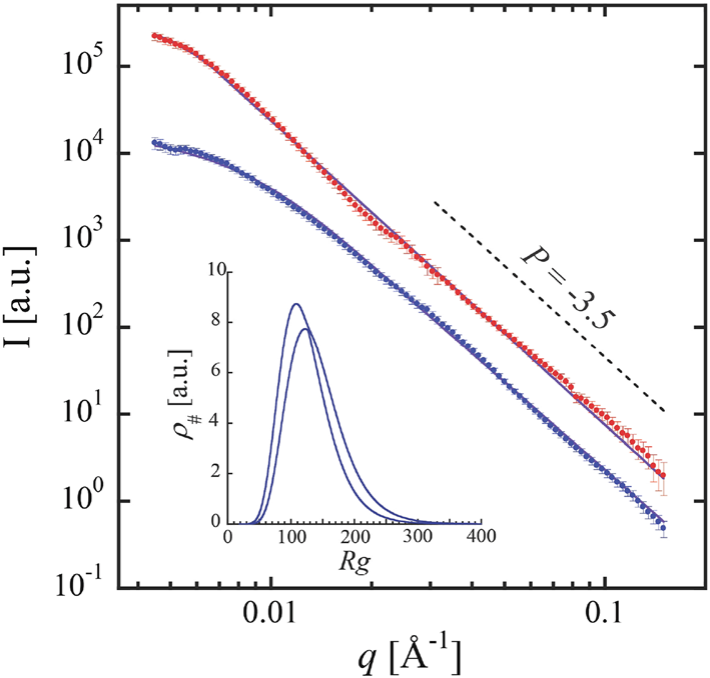
SAXS data for HDPE decomposition products formed at 11.5 ± 5.3 GPa (red circles) and 29.0 ± 1.5 GPa (blue circles) and corresponding fits to Guinier-Porod models (solid lines). The dashed line shows a *P* = − 3.5 power law for reference. A size distribution of particles corresponding to the 29.0 GPa fit is shown in the inset with lines defining the range of distributions within the parameter errors.

To determine the length scales associated with the HDPE decomposition products, complementary small angle x-ray scattering (SAXS) measurements were performed. The dominant SAXS signal from the products likely originates from electron density contrast between solid carbon and a hydrocarbon matrix. Shown in Fig. 4 are 1-D SAXS profiles corresponding to the products obtained from HDPE decomposition at 14.5 ± 5.3 GPa (red) and at 29.0 ± 1.5 GPa (blue). Qualitative examination of the SAXS data reveals Guinier features at low-*q* corresponding to length scales associated with the solid products and a primarily *P* = -3.5 power-law dependence of the scattering at high-*q* (Fig. 4, dashed line).

An empirical Guinier-Porod model was fit to the SAXS data to provide quantitative information on the size and morphology of the decomposition products. For the products formed at 11.5 ± 5.3 GPa, SAXS measurements were performed both at the decomposition pressure (Fig. 4, red data points) and after releasing to ambient pressure (~ 0 GPa). Solid lines are fits of the Guinier-Porod model to the 11.5 GPa measurement indicating a ~ 33 nm length scale in the products (*R*_*g*_ = 32.7 ± 2.1 nm) and a high-q power law of *P* = − 3.45 ± 0.05 potentially indicative of rough surfaced particles. Assuming a spherical morphology, the modelled *R*_*g*_ corresponds to a particle diameter of *D* = 84.4 ± 5.4 nm. After releasing to approximately ambient pressure, there were insignificant changes to the SAXS signal and the *R*_*g*_ and *P* model parameters remained the same within errors. Notably, XRD did not detect crystalline hydrocarbons after pressure release suggesting that the hydrocarbon products are in a fluid state and supporting the interpretation that solid carbon products dominate the SAXS signal. In these cases, including polydispersity of the product length scale did not significantly improve the fits.

Applying a monodisperse Guinier-Porod model to describe the SAXS from the products formed and measured at 29.0 ± 1.5 GPa (Fig. 4, blue data points) yielded a product length scale of *R*_*g*_ = 18.5 ± 1.4 nm. However, the fit to the data was significantly improved by including polydispersity in the form of a log-normal distribution of Guinier-Porod contributions where the width of the distribution was correlated to the mean based on kinetic coagulation models^30^. This approach yielded a comparable mean length scale for the products of $\overline{Rg}$ = 13.4 ± 0.8 nm, corresponding to spheres of $\overline{D}$ = 34.6 ± 2.1 nm, with the range of distributions associated with the errors shown as an inset in Fig. 4. Again, the SAXS signal was largely independent of pressure. After dropping the pressure to 7.3 ± 3.6 GPa, the change in the fit parameters was smaller than the estimated parameter errors.

## Discussion

We provide experimental evidence for the formation of a nanodiamond and methane containing product mixture formed from the decomposition of HDPE at high pressures (11.5 and 29.0 GPa) and temperatures in excess of 2500 K. After approximately one second at the high P–T conditions, the material was quenched to ambient temperature and synchrotron x-rays were used to measure diffraction and small angle scattering at pressures ranging from ambient to 29 GPa. No signature of the initial HDPE structure was observed indicating decomposition of the polymer into reaction products. XRD identified cubic diamond and methane as the predominant crystalline product species.

Length scales associated with the diamond products were estimated from Guinier features in the SAXS data and from diffraction peak widths using the Scherrer formula, with good agreement between values obtained by each approach. Assuming spherical particles, the average nanodiamond diameters were approximately 80 nm for products formed from HDPE decomposition at 11.5 GPa and 35 nm for products formed from decomposition at 29.0 GPa. Despite formation at comparable P–T conditions, these sizes are roughly an order of magnitude larger than typical 4–5 nm diameter detonation nanodiamonds^31^. This difference likely results from both the sub-microsecond duration of the detonation reaction zone, providing a shorter window for diffusion limited carbon clustering compared to the conditions studied here, and the oxygen content of high explosives, providing a competing transformation pathway to oxidized carbon forms.

As a function of pressure, the molecular volume of methane products matched the methane EOS within experimental uncertainty and the observed phase transitions were consistent with the high pressure methane phase diagram. The pressure–volume relationship of the diamond products exhibited a strong agreement with the bulk diamond EOS but with slightly greater values for the atomic volume (V_0_) and bulk modulus (K_0_) at ambient conditions. Comparable variation in V_0_ observed in detonation nanodiamond have been attributed to impurities or defects in the diamond lattice^32^. The small difference in K_0_ relative to bulk diamond may be attributed to finite size effects stemming from significant contributions from the surface atoms of the nanoparticles. Relative to 444.5 GPa for bulk diamond, the core of nanodiamonds ≤ 5 nm in diameter have a substantially higher bulk modulus between 500 and 560 GPa^33, 34^. The bulk modulus of 474 ± 15 GPa for 35–80 nm diameter nanodiamonds observed here is consistent with an intermediate finite size effect on compressibility.

Here, laser heated DAC experiments enabled polyethylene to be decomposed at near equilibrium high P–T conditions and provided the advantage of measuring the products states at a series of P–T conditions. This allowed XRD to obtain the bulk moduli of the products and to detect crystalline methane, which is fluid under both decomposition and ambient conditions, in the product mixture. While our results provided no means to estimate the relative product fractions, the methane and diamond constituents of the product mixture are in good agreement with thermodynamic and chemical equilibrium predictions^10^. In contrast, polyethylene dynamically compressed to P > 100 GPa and temperatures between 2000 and 4000 K for times on the order of a few nanoseconds did not decompose^12^. This difference suggests that either chemical decomposition occurs at longer time scales or that higher pressure stabilizes the chemical bonds such that a polymeric structure is retained, although the latter is not consistent with thermodynamic and chemical equilibrium predictions. At longer time scales, polyethylene dynamically compressed to 28–40 GPa decomposed for times on the order of hundreds of nanoseconds decomposed to solid carbon, methane, and hydrogen products^11^. However, the solid carbon products were not consistent with diamond forms suggesting that atomic rearrangement occurring at longer time scales may be involved in nanodiamond formation. Such kinetics may be probed by short pulse laser heating and rapid quenching of the decomposition products. Additionally, laser heated DAC experiments with *in-situ* x-ray diagnostics may also provide a means to investigate kinetics, at time scales of milliseconds to minutes, of nanodiamond growth in the polymer decomposition products.

## Methods

HDPE was purchased from Polymer Industries (Densetec grade), with a density of 0.957 g/cm^3^ and ~ 78% crystallinity. High pressures were achieved using a symmetric diamond anvil cell (DAC) equipped with type II diamonds with 300 µm diameter culets and a rhenium gasket. Gaskets were preindented to ~ 100 µm and 100 µm diameter holes were drilled to form the sample chamber. Small flakes of HDPE were loaded into the sample chamber along with ruby balls used as an internal pressure standard. Pressures were determined ex-situ by ruby fluorescence and in-situ by ruby diffraction^35, 36^. Differences in the pressures obtained by the two methods was relatively large indicative of possible annealing of non-hydrostatic stresses after laser heating and pressure gradients within the DAC sample chamber. Under the assumption that the in-situ method is more representative of the sample state probed by the x-ray measurements, pressures reported here were obtained from ruby diffraction with uncertainty corresponding to the difference in pressure values obtained by the two approaches.

### CO_2_ laser heating

A Synrad Firestar t60 CO_2_ laser was used to perform on-axis laser heating along the compression axis of the DAC and normal to the diamond table surface. The optical layout consisted of two parts: CO_2_ laser delivery, and visible/near-IR imaging and spectroscopy. Controlled by direct modulation of the CO_2_ laser power, the laser was capable of delivering 80 W with a 10.6 µm wavelength with the laser light focused into the DAC by an aspheric ZnSe with f = 25 mm at 10.6 mm. The laser heating protocol involved gradual increase of laser power. Before reaction, no black body radiation signal was observed indicating temperature < 1200 K. Reaction of the sample lasted for approximately one second and was coincident with black body radiation from the sample that saturated the visualization camera, consistent with temperatures between 2500 and 4500 K.

### X-ray diffraction (XRD) measurements

X-ray measurements were performed on the HP-CAT beamline 16-ID-D at the Advanced Photon Source (APS), Argonne National Laboratory (ANL). Synchrotron x-rays were monochromated to an energy of 20 keV, corresponding to a wavelength λ = 0.6199 Å, and reflection from Pt (in the vertical direction) and Rh (in the horizontal direction) KB mirrors was used to remove higher energy harmonics and focus the x-ray beam between the sample and SAXS detector positions resulting in a beam size of ~ 25 × 25 µm (FWHM) at the sample position and ~ 500 × 500 µm (FWHM) at the SAXS detector position. A Pilatus 100 k detector was used to measure XRD and a MAR345 detector was used to measure SAXS. The XRD detector was calibrated using CeO_2_ diffraction, offset to enable the SAXS to pass, and positioned 185 mm from the sample to capture a momentum transfer of 1.5 < *q* < 3.6 Å^−1^ with azimuthal coverage of ~ 40–120°. XRD data was radially integrated and a polynomial background was subtracted to obtain 1-D diffraction patterns using the DIOPTAS software packaged^37^. Diffraction peaks were fit using a pseudo-Voigt function consisting of a linear combination of a Gaussian and Lorentzian distribution. Uncertainty in the peak fitting parameters were estimated by incrementing the parameter of interest while allowing all others to fit until *χ*^2^ increased by 10% from the minimum value.

### Small angle x-ray scattering (SAXS) measurements

The SAXS detector, with a 345 mm diameter active area, was positioned 3030 mm downstream from the sample and calibrated using diffraction from a silver behenate standard. To minimize air scattering, a He filled flight path was installed with a 50 µm thick mica window upstream and a Kapton window downstream to transmit the x-ray beam. A 2 mm diameter tungsten beam stop, positioned immediately upstream of the Kapton window, was used to block the direct beam. Additionally, a 17.91 mm diameter 91.7 wt% Cu 8.3 wt% Ni (U.S. dime) beam stop was translated in or out of the beam path to block both the direct beam and intense low q scattering signals. Due to dynamic range limitations of the MAR345 detector, SAXS data was collected in two measurements with overlapping *q* ranges. The low *q* configuration employed only the 2 mm diameter tungsten beam stop, capturing data down to *q* = 0.0025 Å^−1^ but with poor signal-to-noise above *q* = 0.05 Å^−1^. For the high *q* configuration, the 17.91 mm diameter beam stop additionally blocked the high intensity low *q* scattering and measured SAXS intensity from 0.03 < *q* < 0.5 Å^−1^. In practice, the low *q* range was limited to *q* = 0.0045 Å^−1^ by artifacts originating from intense diamond diffraction and the SAXS signal was limited to a maximum of *q* = 0.15 Å^−1^ by background. SAXS measurements through an assembled DAC not containing a sample (*i.e.* opposing diamonds and Re gasket) were performed to quantify the diamond, window, air and other instrument specific scattering signals. The SAXS signal from the sample, in arbitrary units of intensity, was obtained by applying a dark field correction, subtracting the intensity of the empty DAC measurements, radially integrating the detector images, and scaling the two measurement ranges to each other in the overlap region. Error bars on the data points represent a combination of statistical errors and estimated 5% systematic errors.

An empirical Guinier-Porod model was fit to the SAXS data, where the intensity as a function of momentum transfer, *q*, was defined as:

1 $$I\left( q \right) = G exp\left( {\frac{{ - q^{2} R_{g}^{2} }}{3}} \right)\quad {\text{for}}\quad q \le q_{1}$$

2 $$I\left( q \right) = \frac{B}{q^{P}}\quad {\text{for}}\quad q \ge q_{1}$$

where *R*_*g*_ is the radius of gyration, *G* is the Guinier scaling factor, *B* is the Porod scaling factor, and *P* is the Porod exponent. Constraining the values of these terms and their derivatives to be continuous at *Q*_*1*_ results in the relationships:

3 $$q_{1} = Rg^{ - 1} \sqrt{\frac{3P}{2}}$$

4 $$B = G exp\left( { - \frac{P}{2}} \right)\left( \frac{3P}{2} \right)^{\frac{P}{2}} \frac{1}{{Rg^{P} }}$$

thereby eliminating *B* and *q*_*1*_ as fitting parameters^38^. A Levenburg-Marquardt minimization algorithm was used to vary the model parameters in order to obtain the solution corresponding to the lowest *χ*^2^ goodness-of-fit. Parameter errors were estimated by incrementing the parameter of interest while allowing all others to fit until *χ*^2^ increased by 10% from the minimum value.
